# Application of Blockchain Technology in Healthcare: A Comprehensive Study

**DOI:** 10.1007/978-3-030-51517-1_23

**Published:** 2020-05-31

**Authors:** Rim Ben Fekih, Mariam Lahami

**Affiliations:** 8 Digital Research Centre of Sfax, Sfax, Tunisia; 9 Institut Mines-Télécom, CNRS, Paris, France; 10 Université de Sherbrooke, Sherbrooke, QC Canada; 11 Digital Research Centre of Sfax, Sfax, Tunisia; 12 University of Sfax, Sfax, Tunisia; 13 ReDCAD Laboratory, National School of Engineers of Sfax, University of Sfax, BP 1173, 3038 Sfax, Tunisia; 14 Digital Research Center of Sfax, B.P. 275, Sakiet Ezzit, 3021 Sfax, Tunisia

**Keywords:** Blockchain, Healthcare, Review

## Abstract

Blockchain technology has been emerged in the last decade and has gained a lot of interests from several sectors such as finance, government, energy, health, etc. This paper gives a broad ranging survey of the application of blockchain in healthcare domain. In fact, the ongoing research in this area is evolving rapidly. Therefore, we have identified several use cases in the state of art applying the blockchain technology, for instance for sharing electronic medical records, for remote patient monitoring, for drug supply chain, etc. We have focused also on identifying limitations of studied approaches and finally we have discussed some open research issues and the areas of future research.

## Introduction

In the last decade, blockchain is emerging as one of the most promising technology that captures attentions of several academic researches and industry. This concept was originally introduced by Satoshi Nakamoto in a white paper in 2008 [19]. It is defined as a decentralized, distributed, immutable ledger which is used to securely record transactions across many computers in a peer-to-peer network, without the need of third party.

The first generation of blockchain, Blockchain 1.0, is underlying on Bitcoin [19] which is the first implementation of blockchain based on cryptocurrency applications^1^. The next generation, called Block chain 2.0, is emerged with the concept of smart contract that it is considered as a piece of code defined, executed and recorded in the distributed ledger. The third generation of blockchain technology, Blockchain 3.0, deals essentially with non financial applications such as government, energy, health, etc. In fact, several organisations have adopted this technology and applied it for several use cases in the healthcare domain. The most interesting features in blockchain that are beneficial to healthcare applications is decentralization, privacy and security since blockchain technology may ensure for example a secure access to medical data for patients and various stakeholders (insurance companies, hospitals, doctors, etc.).

In this survey, we present the most relevant researches applying blockchain in healthcare sector. The studied approaches are classified according to a wide range of use cases such as electronic medical records [2, 6–8, 16, 22, 25], remote patient monitoring [11, 14, 21], pharmaceutical supply chain [4, 5, 12, 15, 20] and health insurance claims [10, 26]. Additionally, this study discusses the applicability of these solutions and their technical limitations. Moreover, lessons learnt and some research directions are identified.

The remainder of this paper is organized as follows. Section 2 introduces the key concepts to understand blockchain technology. In Sect. 3, we provide some medical uses cases in healthcare that use this promising technology. At the end of this section, we will sum up the main results. In Sect. 4, research challenges and opportunities are highlighted. Finally, Sect. 5 concludes the paper and gives suggestions for future work.

## Key Concepts on Blockchain

In this section, we discuss the core features of blockchain technology to help understanding the rest of this paper.

### Overview and Architecture of Blockchain

Essentially, blockchain is a peer-to-peer network that sits on top of the internet [13], which was introduced in 2008 as part of a proposal for Bitcoin [19]. The blockchain is a public ledger made up of a sequence of blocks, which holds a full history of transaction records that occurred within the network. A block is consisted essentially by a header and a body. The header of each block contains the hash of the previous block. Therefore, the blocks form a chain or a linked list where each block structure is based on the previous one.

Block headers also contain a *timestamp* indicating the time of when the block was published, *a nonce*, which is an arbitrary number that miners would change frequently to get a certain hash value to solve a mathematical puzzle and a *Merkle tree* that fundamentally decreases the exertion required to check transactions inside a block.

A Blockchain transaction can be defined as a small unit of task that is stored in public blocks. Each transaction is verified by consensus of a majority of the system participants. This way, tamperproof is ensured once transactions are packed into the blockchain. In regards to blockchain immutability, a same copy of the ledger is replicated, hosted and maintained by all participants [13].

Regardless of the type of blockchain, the business logic is encoded using smart contracts, a self-executing code on the blockchain framework that allow for straight-through processing. When embedded in the blockchain, smart contracts becomes permanently *tamper-proof*, as no one can change what’s been programmed, *self-verifying* due to automated possibilities and *self-enforcing* when the rules are met at all stages.

Among the important features of Blockchain, decentralization by making the ledger accessible by all participants, immutability, so blockchain is nearly impossible to tamper and is censorship-resistant, availability by providing all peers a copy of the blockchain to get access all timestamped transaction records, and anonymity, where each user can interact with the blockchain with a generated address, that does not reveal the real identity of the user.

### Taxonomy of Blockchain Systems

Current blockchain systems are categorized into four types: public, private, consortium and hybrid blockchains [21].

- **Public Blockchains**: Public blockchains provide a fully decentralized network, where every member can access the blockchain content and could take part in the consensus process (e.g. Bitcoin and Ethereum [23]).
- **Private Blockchains**: Private blockchains are dedicated for single enterprise solutions and utilized to keep track of data exchanges occurring between different departments or individuals. Every participant need consent to join the network and considered as a known member once it has been adhered.
- **Consortium Blockchains**: A consortium blockchain is a permissioned network and public only to a privileged group. It is used as an auditable and reliably synchronized distributed database that keeps track of participant’s data exchanges.
- **Hybrid Blockchains**: Hybrid blockchains combine the benefits of private and public blockchains. Therefore, a public blockchain is employed to make the ledger fully accessible, with a private blockchain running in the background that can control access to the modifications in the ledger.

## Blockchain Use Cases in Healthcare

One of the fields where blockchain is considered to have great potential is healthcare. Understanding the pertinence and importance of blockchain in healthcare, in 2016, the Office of the National Coordinator for Health Information Technology (ONC), composed an ideation challenge for requesting white papers on the potential utilization of blockchain in healthcare. This challenge brought about a few proposed healthcare applications for blockchain.

In this section, we focus on the most important studies classified by several use cases such as electronic medical records, remote patient monitoring, pharmaceutical supply chain and health insurance claims.

### Electronic Medical Records

To transform healthcare, the focus should be attributed to the management of health data that could be improved from the potential to connect heterogeneous systems and increase Electronic Health Records (EHRs) accuracy. While Electronic medical records (EMRs) and EHRs are used interchangeably, there is a difference between the two terms. EMRs term came along first, which is a digital version of the paper charts in the clinician’s office. An EMR contains the medical and treatment history of the patients in one practice. However, EHRs focus on the total health of the patient-going beyond standard clinical data collected in the provider’s office and inclusive of a broader view on a patient’s care [1].

From the mapping study, blockchain technology supports the management of EHRs. In this context, Ekblaw et al. present [7] MedRec, an EHR-related implementation that proposes a decentralized approach to manage authorization, permissions, and data sharing between healthcare stakeholders. MedRec uses ethereum platform to enable patients to have knowledge and information on who can get to their healthcare information.

A second application that integrates EHR, is FHIRChain (Fast Health Interoperability Records + Blockchain) [25]. It’s a blockchain-based application implemented using ethereum for sharing clinical data that focuses on healthcare record management. FHIRChain provides solutions for patients that meet the requirements from the ONC.

Similarly, Xia et al. present Medshare [24] an ethereum application for systems that struggle with a lack of collaboration for sharing data between cloud services due to the adverse risks towards displaying the contents of private data. Medshare provides data provenance, auditing, and control between big data entities for sharing medical data in cloud repositories.

Other blockchain-based EMR applications include MedBlock [8] and BlockHIE [16]. MedBlock [8] provides a mechanism for a record search. The proposed system maintains the address of blocks containing the records of a patient, grouped by a healthcare provider or department. Each patient inventory contains a reference to the corresponding record on the blockchain. BlocHIE [16] proposed by Jiang et al. where they present a healthcare platform based on blockchain technology.

To keep exploiting existing databases, BlocHIE combines both off-chain storage, where data is stored in external hospitals’ databases, and on-chain verification. The blockchain system stores a hashed value of external records. Authors improve fairness and throughput by proposing FAIR-FIRST and TP&FAIR, two fairness-based transaction packing algorithms. There is also another healthcare blockchain-based framework, Ancile [6] which uses ethereum smart contracts to achieve data privacy, security, access control and interoperability of EMRs.

Roehrs et al. [22] present omniPHR, a distributed model that maintain an interoperable single-view of Personal Health Records (PHR). The proposed solution is based on an elastic, interoperable and scalable architecture of PHR data. Furthermore, omniPHR evaluation could ensure the division of PHR into data blocks and its distribution in a routing overlay network.

### Remote Patient Monitoring

To be able to remotely monitor the status of the patient, remote patient monitoring covers the collection of medical data through mobile devices, body area sensors and IoT (Internet of Things) devices. Blockchain play an important role in storing, sharing and retrieving the remotely collected biomedical data.

In this context, Ichikawa et al. [14] present an application where mobile devices are used to transmit data to a blockchain-based application on Hyperledger Fabric.

Griggs et al. [11] demonstrate how Ethereum smart contracts provides automated interventions in a secure environment by supporting real-time patient monitoring application. Other proposed approaches present the great potentials of Internet of Things (IoT) in many domains, especially it’s being heavily exploited and used in e-health. In this direction, Ray et al. propose IoBHealth [21], a data-flow architecture that combines the IoT with blockchain and can be used for accessing, storing and managing of e-health data.

### Pharmaceutical Supply Chain

One other identified use case of blockchain is in the pharmaceutical industry. The delivery of counterfeit or inadequate medications can have critical consequences for the patients. Blockchain technology has been identified as having the capability to address this problem.

Bocek et al. [4] present Modum.io AG, a startup that uses blockchain to achieve data immutability. To verify the compliance to quality control temperature requirements, this startup creates public accessibility of the temperature records of pharmaceutical products during their transportation.

Counterfeit drugs also have been addressed by [5, 12, 20] where authors prevent counterfeiting by proposing a secure, immutable and traceable pharmaceutical supply chain based on blockchain technology.

With regards to drug regulations issues, Jamil et al. [15] addressed drugs standardization problems. Authors have highlighted the difficulties to detect falsified drugs and proposed a blockchain-based solution to detect counterfeits.

While there is a minority of papers that present an implementation of the proposed system, some interesting reviews discuss pharmaceutical supply chain issues [9, 17].

### Health Insurance Claims

Health Insurance claims are one of healthcare fields that can benefit from blockchain’s immutability, transparency and auditability of data stored on it.

While Healthcare insurance claim processing is an important area where blockchain has potentials [10]. However, prototypes implementations of such systems are very limited. We can find MIStore [26], a blockchain-based medical insurance system that provides medical insurance industry with encrypted and immutably stored medical insurance data.

We present a summary of the studied papers in Table 1. We have noticed that the majority of these applications are developed on popular blockchain frameworks, such as Ethereum and Hyperledger Fabric.

**Table 1.** Major contributions classified by use cases

Use cases	Paper	Framework	Data Storage	Contribution	Year
Electronic medical records	[7]	Ethereum	Off-chain	Provides a patient-centric system for a transparent and accessible view of medical history	2016
	[24]	Ethereum	Off-chain	Propose a platform for shared medical data in cloud repositories	2017
	[22]	Specific	Off-chain	A PHR distributed model that propose solutions for latency issues	2017
	[25]	Ethereum	Hybrid	Proposes a blockchain-based EMR application that meets ONC requirements	2018
	[8]	Proprietary	Off-chain	Provides a blockchain-based EMR management system	2018
	[16]	Proprietary	Off-chain	A healthcare system that Combines both off-chain storage and on-chain verification	2018
	[6]	Ethereum	Hybrid	Proposes an electronic health records system that protects personal health information	2018
Remote patient monitoring	[14]	Hyperledger Fabric	Off-chain	A mobile Health blockchain-based system for cognitive behavioral therapy for insomnia	2017
	[11]	Ethereum	Hybrid	Proposes to use blockchain-based smart contracts to perform real-time data analysis	2018
	[21]	–	–	Propose an architecture that integrates blockchain and IoT sensory data collected from patients	2020
Pharmaceutical supply chain	[4]	Ethereum	Off-chain	Maintains public temperature records’ accessibility of drugs during their transportation	2017
	[12]	–	–	Explaines blockchain usability to add traceability and visibility to drugs supply	2018
	[5]	Hyperledger Fabric	On-chain	Design a blockchain-based control system for the control of drugs turnover	2019
	[20]	Hyperledger Fabric	On-chain	Design a secure, immutable and traceable pharma supply chain	2019
Health insurance claims	[26]	Ethereum	On-chain	Proposes a blockchain-based medical insurance storage system	2018

## Research Challenges and Opportunities

Based on the proposed prototypes and developed applications, we can identify different limitations of the healthcare Blockchain-Based applications.

First, EMR systems do not address semantic interoperability [3]. Consequently, manual inspection and mapping of predefined ontologies from medical and health data experts are required. Second, clinical malpractice cannot be controlled at this level. Moreover, scalability and interoperability issues represent the main focus of current and future studies in this field. Interoperability challenge reveals the fact of missing standards for developing healthcare applications based on blockchain technology. Thus, the different developed applications may not be able to interoperate. In addition, scalability is a major issue in blockchain-based healthcare systems [18] especially towards the volume of medical data involved. Due to high-volume healthcare data, it is not practicable to store it on-chain i.e. on blockchain, as this is may lead to serious performance degradation. Furthermore, there is a problem of latency caused by the speed of transactions’ processing and off-chain data load in a blockchain-based system. Finally, another weakness is related to blockchain immutability and self-execution of code, since smart contracts could become vulnerable to hackers. Just between 2016 and 2018, attacks such as the decentralized autonomous organization (DAO) attack cause a loss of millions of dollars as part of the assets held by the smart contracts.

## Conclusion

The present study gave an overview about the application of Blockchain in Healthcare. In fact, due to the exponential growth of this technology, blockchain has been applied in several use cases with the aim of enhancing the automation of medical services.

Our study shows that the majority of researches applying blockchain in healthcare are concentrated towards sharing Electronic Health Records. Other investigations should be considered by blockchain researchers in domains such as biomedical research, pharmaceutical supply chain, insurance. Furthermore, we noticed that rarely are papers dealing with implementation details.

Even though, blockchain technology offers promising features, there is still a need for more research to better understand, efficiently and securely develop and evaluate this technology. Ongoing efforts have been conducted to overcome limitations in scalability, security and privacy in order to improve stakeholders’ confidence in using this technology and to increase its adoption in healthcare.

## Footnotes

1 Other blockchain 1.0 technologies have been appeared such as Dash, Litecoin, etc.
